# Trichloroethanol, an active metabolite of chloral hydrate, modulates tetrodotoxin-resistant Na^+^ channels in rat nociceptive neurons

**DOI:** 10.1186/s12871-023-02105-0

**Published:** 2023-04-29

**Authors:** Gimin Kim, Hyunjung Kim, Il-Sung Jang

**Affiliations:** 1 Department of Pediatric Dentistry, School of Dentistry, Kyungpook National University, Daegu, 41940 Republic of Korea; 2 Department of Pharmacology, School of Dentistry, Kyungpook National University, 2177 Dalgubeol-daero, Jung-gu, Daegu, 41940 Republic of Korea; 3 Brain Science & Engineering Institute, Kyungpook National University, Daegu, 41940 Republic of Korea

**Keywords:** Chloral hydrate, Trichloroethanol, Analgesia, TTX-R Na^+^ channels, Nociceptive neurons, Patch clamp

## Abstract

**Background:**

Chloral hydrate is a sedative-hypnotic drug widely used for relieving fear and anxiety in pediatric patients. However, mechanisms underlying the chloral hydrate-mediated analgesic action remain unexplored. Therefore, we investigated the effect of 2′,2′,2′-trichloroethanol (TCE), the active metabolite of chloral hydrate, on tetrodotoxin-resistant (TTX-R) Na^+^ channels expressed in nociceptive sensory neurons.

**Methods:**

The TTX-R Na^+^ current (I_Na_) was recorded from acutely isolated rat trigeminal ganglion neurons using the whole-cell patch-clamp technique.

**Results:**

Trichloroethanol decreased the peak amplitude of transient TTX-R I_Na_ in a concentration-dependent manner and potently inhibited persistent components of transient TTX-R I_Na_ and slow voltage-ramp-induced I_Na_ at clinically relevant concentrations. Trichloroethanol exerted multiple effects on various properties of TTX-R Na^+^ channels; it (1) induced a hyperpolarizing shift on the steady-state fast inactivation relationship, (2) increased use-dependent inhibition, (3) accelerated the onset of inactivation, and (4) retarded the recovery of inactivated TTX-R Na^+^ channels. Under current-clamp conditions, TCE increased the threshold for the generation of action potentials, as well as decreased the number of action potentials elicited by depolarizing current stimuli.

**Conclusions:**

Our findings suggest that chloral hydrate, through its active metabolite TCE, inhibits TTX-R I_Na_ and modulates various properties of these channels, resulting in the decreased excitability of nociceptive neurons. These pharmacological characteristics provide novel insights into the analgesic efficacy exerted by chloral hydrate.

## Background

Nociceptive signals generated at peripheral tissues are transmitted to the central nervous system through the generation and conduction of action potentials. Thus, various voltage-gated ion channels, such as voltage-gated Na^+^ and K^+^ channels, expressed in sensory neurons play pivotal roles in nociceptive transmission. Among the nine types of voltage-gated Na^+^ channels, tetrodotoxin-sensitive (TTX-S) Na_V_1.7 and TTX-resistant (TTX-R) Na_V_1.8 and Na_V_1.9 are specifically expressed in nociceptive sensory neurons within the dorsal root ganglia (DRG) and trigeminal ganglia (TG) [1]. In particular, Na_V_1.8 plays a pivotal role in the generation and conduction of action potentials in response to sustained nociceptive signals, as this channel remains activated even at relatively depolarized membrane potentials [2, 3]. Na_V_1.8 has also been implicated in the development and maintenance of inflammatory hyperalgesia [4]. We reported that the TTX-R Na^+^ channels (Na_V_1.8)-mediated persistent Na^+^ current (I_NaP_), which is a non-inactivating current during sustained depolarizing stimuli, contributes to the excitability of nociceptive neurons, and the density of TTX-R I_NaP_ is increased by inflammatory mediators [5]. These findings suggest that TTX-R Na^+^ channels and TTX-R I_NaP_ mediated by the Na_V_1.8 subtype are potential pharmacological targets for relieving inflammatory pain [1].

Children are mentally, physically, and emotionally immature, and those visiting the dentist for the first time have heightened fear and anxiety about dental treatment. Although various psychological and physical methods, such as voice control and mouth covering, can be used to control behavioral states, such methods are not always successful. Therefore, behavioral control using sedative and anesthetic agents, such as chloral hydrate, midazolam, and nitrous oxide, is recommended for the dental treatment of pediatric patients [6].

Chloral hydrate, a first-generation sedative-hypnotic drug, is used for sedation and for relieving fear and anxiety in pediatric patients undergoing medical procedures [7, 8], such as dental treatment [6, 7, 9]; however, its use in humans or animals has been declining [10–12]. Chloral hydrate is readily absorbed after oral administration but is undetectable 10 min after intake because of its rapid metabolization by aldehyde dehydrogenase in hepatocytes and erythrocytes [8, 11–13]. Owing to its rapid conversion after oral administration, most pharmacological properties of chloral hydrate have been determined using its active metabolite 2′,2′,2′-trichloroethanol (TCE) [14]. Although the mechanisms underlying the sedative and anesthetic action of chloral hydrate or TCE remain poorly understood, TCE is known to act on GABA_A_ receptors to potentiate Cl^−^ conductance in the central nervous system, similar to benzodiazepines [15]. Notably, TCE potentiates native [15] and recombinant [16] GABA_A_ receptors expressed in mouse hippocampal neurons and HEK 293 cells, respectively. TCE also potentiates recombinant glycine and 5-HT_3_ receptors expressed in *Xenopus* oocytes [17, 18]. In addition, TCE reportedly inhibits excitatory glutamate receptors, such as AMPA and NMDA receptors, in central neurons [19, 20]. Such direct modulation of inhibitory or excitatory receptors might be responsible for the sedative and hypnotic actions mediated by chloral hydrate. While it is not directly related to the anesthetic efficacy, TCE also inhibits mitochondrial ATP-sensitive K^+^ channels in rat cardiac myocytes [21].

In addition to its anesthetic action, chloral hydrate reportedly exhibits analgesic effects [7, 22]. Such analgesic effects might result from the modulation of nociceptive transmission; however, limited information is available regarding the mechanisms underlying the analgesic action of chloral hydrate. Therefore, in this study, we examined the effect of TCE on TTX-R Na^+^ channels in acutely isolated nociceptive neurons. To our knowledge, this is the first study describing the pharmacological role of TCE in peripheral analgesic efficacy.

## Materials and methods

### Preparation

All experiments in this study complied with the guiding principles for the care and use of animals and were approved by the Council of Kyungpook National University (KNU-2019-0053). Every effort was made to minimize the number of animals used and their suffering.

Sprague Dawley rats (postnatal age: 3–4 weeks; both sexes; Samtako, Osan, Korea) were decapitated under ketamine anesthesia (50 mg/kg, intraperitoneal). Their TGs (V_3_ part) were dissected and treated with a standard external solution (150 mM NaCl, 3 mM KCl, 2 mM CaCl_2_, 1 mM MgCl_2_, 10 mM glucose, and 10 mM HEPES, [pH 7.4 with Tris-base]) containing 0.3% collagenase and 0.3% trypsin at 37 °C for 40–50 min. Subsequently, TG neurons were mechanically dissociated by trituration using fire-polished Pasteur pipettes in culture dishes (CELLSTAR® TC, Greiner bio-one, Chonburi, Thailand). Isolated TG neurons were used for electrophysiological recordings 2–6 h after preparation.

### Electrical measurements

Electrical measurement data were collected using conventional whole-cell patch recordings and a standard patch-clamp amplifier (Multiclamp 900B; Molecular Devices, Union City, CA, USA). Neurons were voltage clamped at a holding potential (V_H_) of − 80 mV. Patch pipettes were prepared from borosilicate capillary glass (G-1.5; Narishige, Tokyo, Japan) using a pipette puller (P-97; Sutter Instrument Co., Novato, CA, USA). The resistance of recording pipettes filled with an internal solution (135 mM CsF, 10 mM CsCl, 2 mM EGTA, 2 mM ATP-Na_2_, and 10 mM HEPES [pH 7.2 with Tris-base]) was 0.7–1.0 MΩ. Membrane potentials were corrected for the liquid junction potential, and the pipette capacitance and series resistance (40–70%) were compensated for. Neurons were viewed under phase contrast on an inverted microscope (TE2000; Nikon, Tokyo, Japan). Membrane currents were filtered at 2–5 kHz, digitized at 10–20 kHz, and stored on a computer equipped with pCLAMP 10.7 (Molecular Devices). The bath solution was composed of: 130 mM NaCl, 20 mM tetraethylammonium-Cl, 2 mM CaCl_2_, 1 mM MgCl_2_, 10 mM HEPES, 10 mM glucose, 0.0003 mM TTX, and 0.01 mM CdCl_2_ (pH 7.4 with Tris-base), except where indicated. To record TTX-R I_Na_, capacitative and leakage currents were subtracted using the P/4 subtraction protocol (pCLAMP 10.7). The internal solution used in the current-clamp experiments for recording voltage responses was composed of 135 mM KF, 10 mM KCl, 2 mM EGTA, 2 mM ATP-Na_2_, and 10 mM HEPES (pH 7.2 with Tris-base), whereas the external solution was composed of 150 mM NaCl, 3 mM KCl, 2 mM CaCl_2_, 1 mM MgCl_2_, 10 mM glucose, 10 mM HEPES, and 0.0003 mM TTX (pH 7.4 with Tris-base).

### Data analysis

The peak amplitude, time to peak, and decay time constant of the transient TTX-R I_Na_ were determined using pCLAMP 10.7. In a subset of measurements, the amplitude of TTX-R I_Na_ was transformed into conductance (G) using the following equation:

$$\mathrm G=\mathrm I/\left(\mathrm V-{\mathrm E}_{\mathrm{Na}}\right)$$

The voltage-activation and voltage-inactivation relationships of TTX-R Na^+^ channels were respectively fitted to the following Boltzmann equations:

$$\text{G/G}_{\text{max}}=1/\left(1+\exp\left[\left\{{\mathrm V}_{50,\mathrm{activation}}-\mathrm V\right\}/\mathrm k\right]\right)\;\mathrm{and}\;\mathrm I/\mathrm{I_{max}}=1-1/\left(1+\exp\left[\left\{{\mathrm V}_{50,\mathrm{inactivation}}-\mathrm V\right\}/\mathrm k\right]\right),$$

where G_max_ is the maximum conductance, I_max_ is the maximum current amplitude, V_50, activation_ is the half-maximum voltage for activation, V_50, inactivation_ is the half-maximum voltage for fast inactivation, and *k* is the slope factor. Kinetic data for the onset of inactivation and recovery from inactivation were fitted to the following equations, respectively:

$$\begin{aligned} \mathrm I\left(t\right)={\mathrm A}_0+{\mathrm A}_{\mathrm{fast}}\times\left(\exp\left[-t/{\mathrm\tau}_{\mathrm{fast}}\right]\right)+{\mathrm A}_{\mathrm{slow}}\;\times\;\left(\exp\left[-t/{\mathrm\tau}_{\mathrm{slow}}\right]\right)\;\mathrm{and}\\\mathrm I\left(t\right)={\mathrm A}_0+{\mathrm A}_{\mathrm{fast}}\times\left(1\;-\;\exp\left[-t/\tau_{\mathrm{fast}}\right]\right)+{\mathrm A}_{\mathrm{intermediate}}\;\times\;\left(1-\exp\left[-t/{\mathrm\tau}_{\mathrm{intermediate}}\right]\right)+{\mathrm A}_{\mathrm{slow}}\times\left(1\;-\;\exp\left[-t/{\mathrm\tau}_{\mathrm{slow}}\right]\right)\end{aligned}$$

In these equations, I(*t*) is the amplitude of TTX-R I_Na_ at time *t*, whereas A_fast_, A_intermediate_, and A_slow_ are the amplitude fractions of τ_fast_, τ_intermediate_, and τ_slow_, respectively. The weighted decay time constant (τ_WD_) for the single transient TTX-R I_Na_ or weighted time constant (τ_weighted_) for the onset of inactivation was calculated using the following equation:

$${\mathrm\tau}_{\mathrm{WD}}\;\mathrm{or}\;{\mathrm\tau}_{\mathrm{weighted}}\;=\;\left(\left[{\mathrm\tau}_{\mathrm{fast}}\times{\mathrm A}_{\mathrm{fast}}\right]+\left[{\mathrm\tau}_{\mathrm{slow}}\times{\mathrm A}_{\mathrm{slow}}\right]\right)/\left({\mathrm A}_{\mathrm{fast}}+{\mathrm A}_{\mathrm{slow}}\right)$$

The τ_weighted_ for the recovery from inactivation was calculated using the following equation:

$${\mathrm\tau}_{\mathrm{weighted}}=\left(\left[{\mathrm\tau}_{\mathrm{fast}}\times{\mathrm A}_{\mathrm{fast}}\right]+\left[{\mathrm\tau}_{\mathrm{intermediate}}\times{\mathrm A}_{\mathrm{intermediate}}\right]+\left[{\mathrm\tau}_{\mathrm{slow}}\times{\mathrm A}_{\mathrm{slow}}\right]\right)/\left({\mathrm A}_{\mathrm{fast}}+{\mathrm A}_{\mathrm{intermediate}}+{\mathrm A}_{\mathrm{slow}}\right)$$

Numerical values are provided as the mean ± standard error of the mean (SEM) following normalization to the control value. Significant differences in the mean amplitude were tested with Student’s paired two-tailed *t*-test using absolute rather than normalized values. *P* < 0.05 was considered statistically significant.

### Drugs

The drugs used in this study were collagenase (type I), trypsin (type I), tetrodotoxin (TTX), Na-ATP, CdCl_2_, and 2′,2′,2′-trichloroethanol (TCE) (all from Sigma, USA) and ketamine (Yuhan Co., Seoul, Korea). The extracellular solution containing drugs was applied to patched neurons using the “Y–tube system” for rapid solution exchange [23] at a perfusion rate of 0.5–0.6 mL/min.

## Results

### Effects of TCE on transient TTX-R Na^+^ currents

**Fig. 1:**
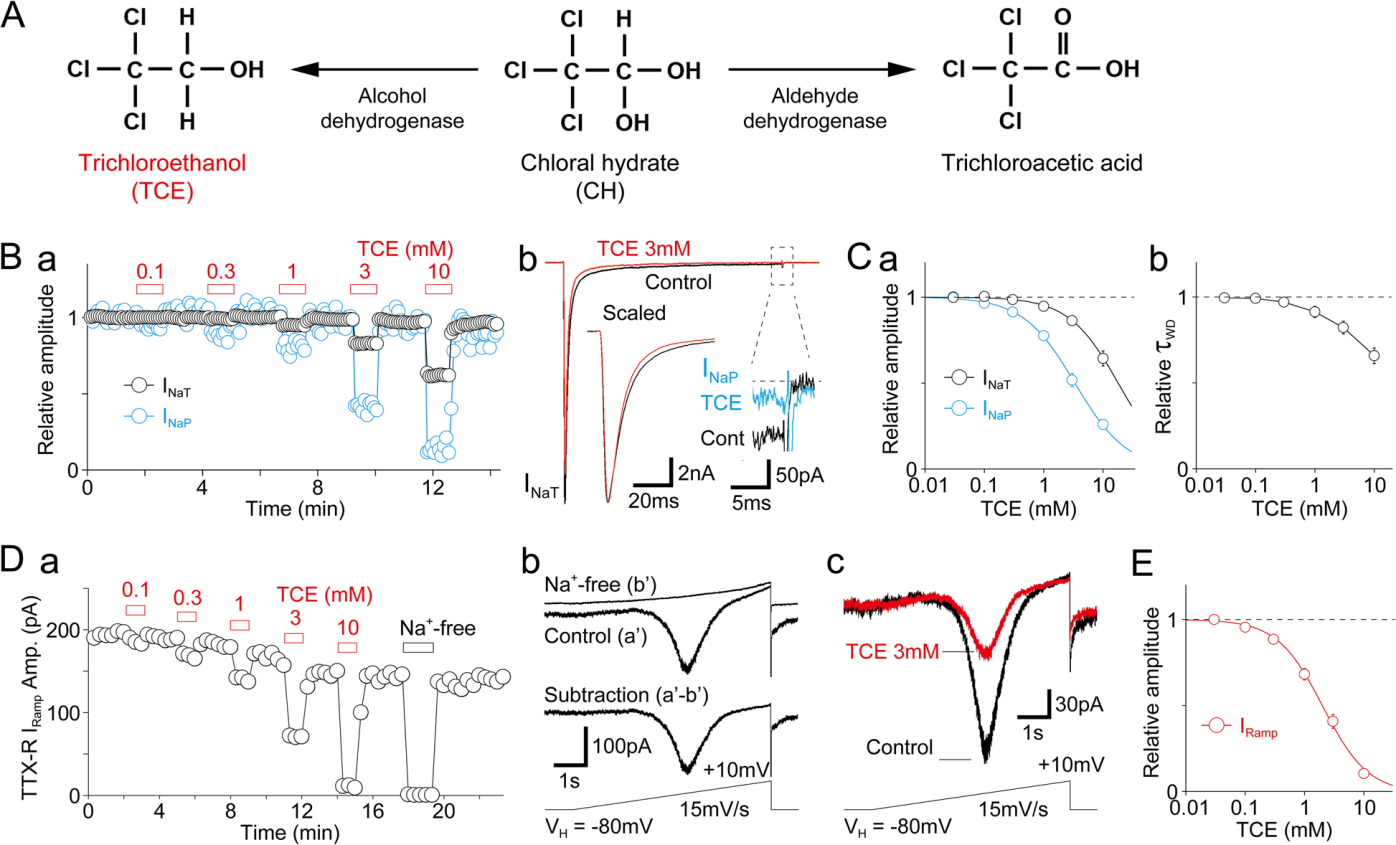
Effect of TCE on TTX-R I_Na_. **(A)** A brief illustration showing chloral hydrate (CH) metabolism after absorption. CH is rapidly converted into trichloroethanol (TCE) and trichloroacetic acid by alcohol dehydrogenase and aldehyde dehydrogenase, respectively. TCE is conjugated with glucuronic acid and excreted through the kidneys. **(B)** **a**, Typical time courses of TTX-R I_Na_ amplitude before and during TCE application to small-sized TG neurons. TTX-R I_Na_ was elicited by electrical stimulation from a V_H_ of − 80 mV to − 10 mV (100 ms duration) at 5 s intervals. The amplitudes of transient (black circles; I_NaT_) and persistent (cyan circles; I_NaP_) TTX-R I_Na_ were normalized to the first amplitude. **b**, Typical traces of TTX-R I_Na_ in the absence and presence of 3 mM TCE. Both I_NaT_ (scaled) and I_NaP_ are presented in expanded time and amplitude scales, respectively (insets). **(C) a**, The concentration-inhibition relationship of TCE for transient (black circles; I_NaT_) and persistent (cyan circles; I_NaP_) TTX-R I_Na_. Continuous lines represent the best fit using the least squares method. Each point represents the mean and SEM from 6–9 experiments. **b**, The concentration-inhibition relationship of TCE for the weighted decay time constant (τ_WD_) of TTX-R I_Na_. Each point represents the mean and SEM from seven experiments. **(D) a**, A typical time course of slow voltage ramp-induced current (TTX-R I_Ramp_) amplitude before and during TCE application to small-sized TG neurons. The TTX-R I_Ramp_ was elicited by slow voltage ramp stimulation from a V_H_ of − 80 mV to + 10 mV (15 mV/s, every 20 s). **b**, Typical traces of TTX-R I_Ramp_ in the presence (a’) and absence (b’) of extracellular Na^+^. Subtraction (a’–b’) of two traces yields TTX-R I_Ramp_ without the capacitative current. **c**, Typical traces of TTX-R I_Ramp_ in the absence and presence of 3 mM TCE. **(E)** The concentration-inhibition relationship of TCE for TTX-R I_Ramp_. The continuous line represents the best fit using the least squares method. Each point represents the mean and SEM from seven experiments

We first examined the effect of TCE, an active metabolite of chloral hydrate (Fig. 1A), on transient TTX-R I_Na_ in nociceptive neurons using a whole-cell patch-clamp technique. Small-sized DRG neurons (< 30 μm in diameter; 25.0 ± 4.7 pF; *n* = 65 neurons) were held at a V_H_ of − 80 mV, and brief depolarizing step pulses (up to − 10 mV, 100-ms duration) were applied to elicit the TTX-R I_Na_. Under these conditions, the recorded TTX-R I_Na_ was stable, and TCE inhibited the peak amplitude of the transient component of TTX-R I_Na_ (I_NaT_) in a dose-dependent manner with an IC_50_ value of 18.4 ± 1.1 mM (*n* = 7). In contrast, at a 3 mM concentration, it decreased the amplitude of TTX-R I_NaT_ to 86.3 ± 1.6% of that in the control (*n* = 7, *p* < 0.01; Fig. 1B, Ca). However, TCE more potently inhibited the noninactivating persistent component of transient TTX-R I_Na_ (I_NaP_) in a dose-dependent manner with an IC_50_ value of 3.3 ± 0.4 mM (*n* = 7). In contrast, at a 3 mM concentration it decreased the amplitude of TTX-R I_NaP_ to 51.7 ± 3.9% of that in the control (*n* = 7, *p* < 0.01; Fig. 1B, Ca). In addition, TCE accelerated the decay phase of a single TTX-R I_Na_ in a dose-dependent manner (Fig. 1Cb). Notably, I_NaP_ can be elicited by slow voltage ramp stimuli [24, 25]. Therefore, we further examined the effect of TCE on the slow voltage ramp-induced current (I_Ramp_). We determined that TCE potently inhibited the TTX-R I_Ramp_ in a concentration-dependent manner with an IC_50_ value of 2.0 ± 0.2 mM (*n* = 7), whereas at a 3 mM concentration, it reduced the TTX-R I_Ramp_ amplitude to 40.8 ± 4.0% of that in the control (*n* = 7; *p* < 0.01; Fig. 1D, E).

### Effects of TCE on the voltage-dependence of TTX-R Na^+^ channels

**Fig. 2:**
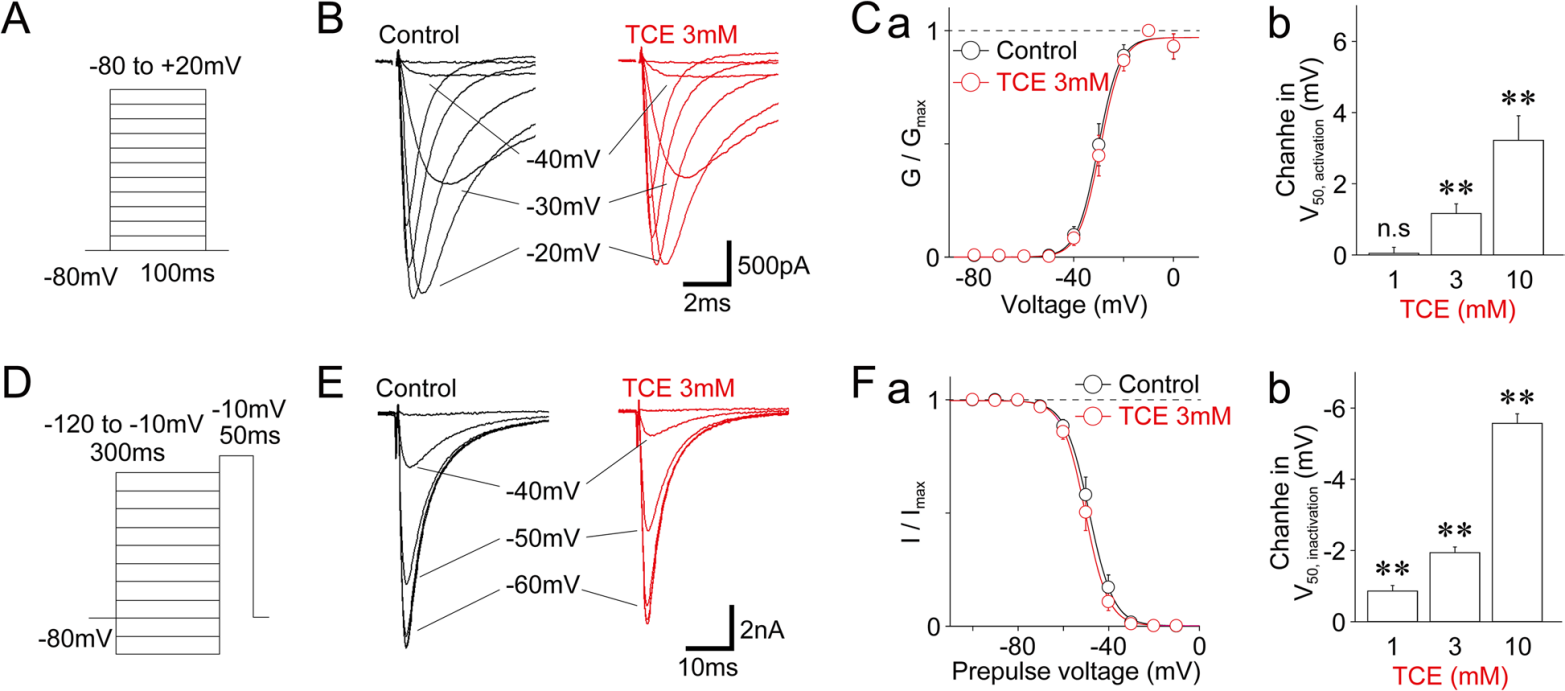
Effect of TCE on the voltage-dependence of TTX-R Na^+^ channels. **(A)** A schematic illustration of voltage step pulses to examine the voltage-activation relationship of TTX-R Na^+^ channels. The TTX-R I_Na_ was induced by 50 ms depolarization pulses from − 80 to + 20 mV in 10 mV increments at a V_H_ of − 80 mV. **(B)** Typical traces of TTX-R I_Na_ elicited by voltage step pulses in the absence (left) and presence (right) of 3 mM TCE. **(C) a**, The conductance-voltage relationship of TTX-R Na^+^ channels in the absence (black circles) and presence (red circles) of 3 mM TCE. Continuous lines represent the best fit of the Boltzmann function. Each point represents the mean and SEM from seven experiments. **b**, TCE-induced changes in the midpoint voltage for the activation (V_50, activation_) of TTX-R Na^+^ channels. Each column represents the mean and SEM from seven experiments for 1, 3, and 10 mM TCE. **; *p* < 0.01, n.s; not significant. **(D)** A schematic illustration of voltage step pulses to examine the voltage-steady state fast inactivation relationship of TTX-R Na^+^ channels. The TTX-R I_Na_ was induced by 50 ms depolarization pulses to − 10 mV after 300 ms prepulse from − 120 to − 20 mV in 10 mV increments. **(E)** Typical traces of TTX-R I_Na_ elicited by voltage step pulses in the absence (left) and presence (right) of 3 mM TCE. **(F) a**, The voltage-inactivation relationship of TTX-R Na^+^ channels in the absence (black circles) and presence (red circles) of 3 mM TCE. Continuous lines represent the best fit of the Boltzmann function. Each point represents the mean and SEM from seven experiments. **b**, TCE-induced changes in the midpoint voltage for the inactivation (V_50, inactivation_) of TTX-R Na^+^ channels. Each column represents the mean and SEM from seven experiments for 1, 3, and 10 mM TCE. **; *p* < 0.01

We then examined the effect of TCE on the voltage-activation relationship of TTX-R Na^+^ channels using depolarizing test pulses (100-ms duration, up to + 20 mV in 10-mV increments; Fig. 2A). Subsequently, TTX-R I_Na_ was converted to conductance, and the normalized conductance values were fitted to the Boltzmann function. TCE shifted the midpoint voltage for activation (V_50, activation_) toward a hyperpolarizing range in a dose-dependent manner. In contrast, at a 3 mM concentration, it changed the V_50, activation_ values by 1.2 ± 0.3 mV (− 29.8 ± 4.4 mV for the control and − 28.6 ± 4.2 mV for TCE treatment; *n* = 7; *p* < 0.01; Fig. 2B, C). Notably, these TCE-induced changes in V_50, activation_ values were dose-dependent (Fig. 2Cb). We also examined the effect of TCE on the steady-state voltage-inactivation relationship of TTX-R Na^+^ channels using depolarization test pulses (100-ms duration, up to − 10 mV after 300-ms prepulse from − 120 to − 10 mV in 10 mV increments; Fig. 2D). When the normalized TTX-R I_Na_ was fitted to the Boltzmann function, TCE shifted the midpoint voltage for inactivation (V_50, inactivation_) toward a hyperpolarizing range in a dose-dependent manner. In contrast, at a 3 mM concentration, it changed the V_50, inactivation_ values by − 1.9 ± 0.2 mV (− 48.3 ± 1.7 mV for the control and − 50.2 ± 1.6 mV for TCE treatment; *n* = 7; *p* < 0.01; Fig. 2E, F). These TCE-induced changes in V_50, inactivation_ values were dose-dependent (Fig. 2Fb).

### Effects of TCE on the use-dependent inhibition of TTX-R Na^+^ channels

**Fig. 3:**
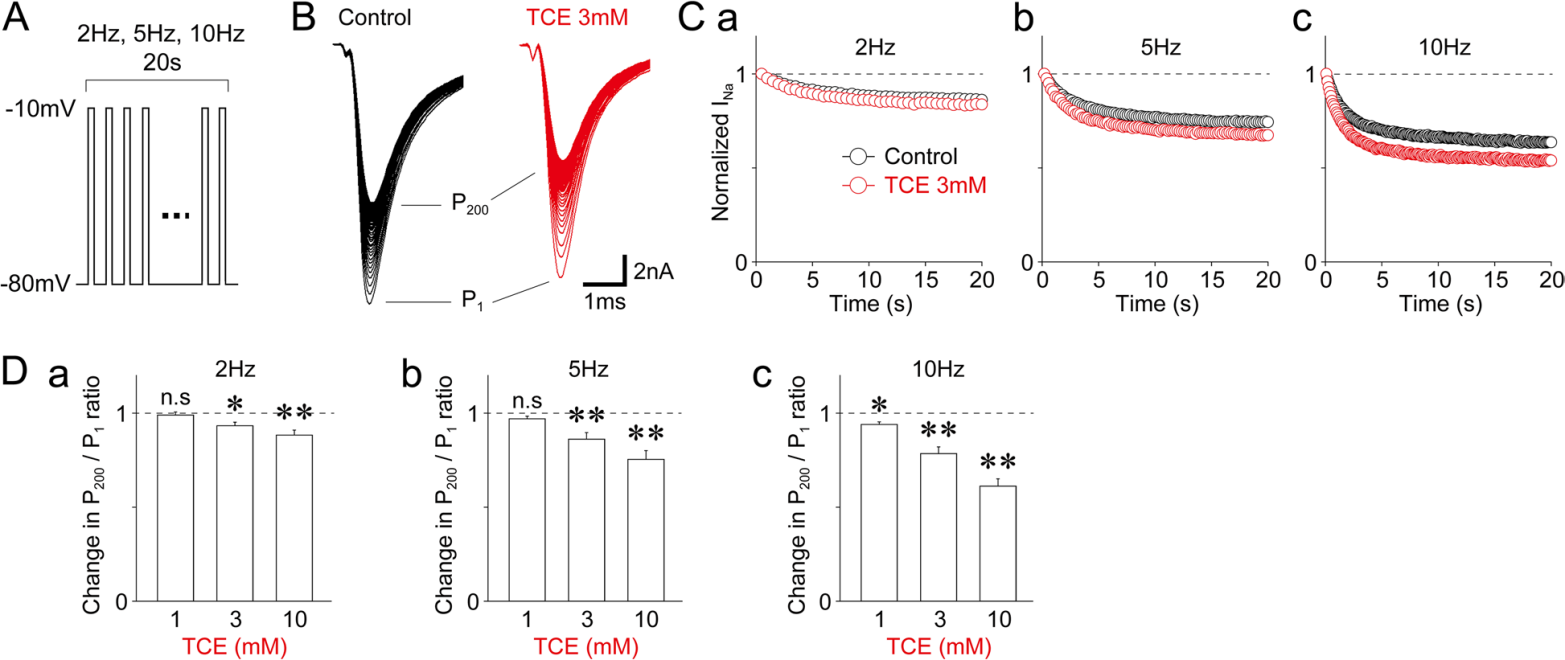
Effect of TCE on the use-dependent inactivation of TTX-R Na^+^ channels. **(A)** A schematic illustration of voltage step pulses to examine the use-dependent inhibition of TTX-R Na^+^ channels. The TTX-R I_Na_ was induced by 40, 100, and 200 successive voltage step pulses (− 80 mV to − 10 mV, 10 ms duration, 2 Hz, 5 Hz, and 10 Hz, respectively). **(B)** Typical traces of TTX-R I_Na_ elicited by 200 successive voltage step pulses (10 Hz) in the absence (left) and presence (right) of 3 mM TCE. **(C)** Time courses of the amplitude of TTX-R I_Na_ during trains of 40 (2 Hz, **a**), 100 (5 Hz, **b**), and 200 pulses (10 Hz, **c**) in the absence (black circles) and presence (red circles) of 3 mM TCE. Each point represents the mean and SEM from eight experiments. **(D)** TCE-induced changes in the P_40_/P_1_ (2 Hz, **a**), P_100_/P_1_ (5 Hz, **b**), and P_200_/P_1_ (10 Hz, **c**) ratios of TTX-R I_Na_. The ratios were normalized to the respective control (dotted lines). Each column represents the mean and SEM from eight experiments for 1, 3, and 10 mM TCE. *; *p* < 0.05, **; *p* < 0.01, n.s; not significant

Next, we examined the effect of TCE on the use-dependence of TTX-R Na^+^ channels using a 20 s series of 40 (2 Hz), 100 (5 Hz), and 200 (10 Hz) depolarizing test pulses (10-ms duration, up to − 10 mV; Fig. 3A). Figure 3B depicts typical TTX-R I_Na_ elicited by 200 (10 Hz) depolarizing test pulses in the absence and presence of 3 mM TCE. Figure 3 C displays typical time courses of TTX-R I_Na_ elicited by 40 (2 Hz), 100 (5 Hz), and 200 (10 Hz) depolarizing test pulses in the absence and presence of 3 mM TCE. When the amplitude ratio of the 40th, 100th, and 200th TTX-R I_Na_ (P_40_, P_100_, and P_200_, respectively) and the first TTX-R I_Na_ (P_1_) was analyzed, TCE decreased the P_40_/P_1_, P_100_/P_1_, and P_200_/P_1_ ratios in a dose-dependent manner (Fig. 3D). In contrast, at a 3 mM concentration, it decreased the P_200_/P_1_ ratio to 78.5 ± 3.6% of that in the control (*n* = 8, *p* < 0.01; Fig. 3Dc).

### Effects of TCE on inactivation and recovery kinetics of TTX-R Na^+^ channels

**Fig. 4:**
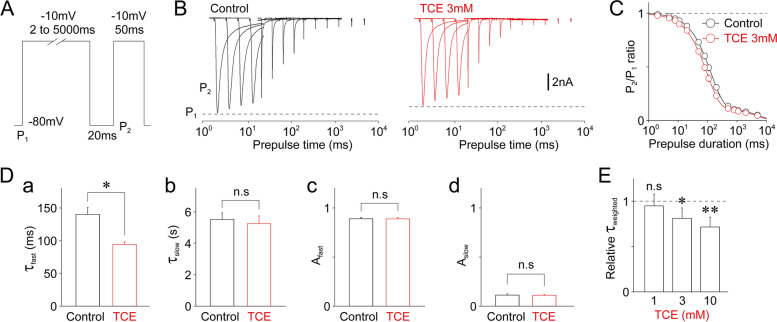
Effect of TCE on the inactivation of TTX-R Na^+^ channels. **(A)** A schematic illustration of the two-pulse protocol for determining inactivation kinetics. The TTX-R I_Na_ was induced by initial conditioning pulses (P_1_: − 10 mV depolarization, 2 to 5000 ms duration), which were followed by test pulses (P_2_: − 10 mV depolarization, 50 ms duration). The second TTX-R I_Na_ was recovered with an interpulse interval of 20 ms at − 80 mV potential. **(B)** Typical traces of TTX-R I_Na_ elicited by test pulses (P_2_) in the two-pulse protocol in the absence (left) and presence (right) of 3 mM TCE. The dotted lines represent the amplitude elicited by the initial conditioning pulses (P_1_). **(C)** The P_2_/P_1_ ratio of TTX-R I_Na_ against the duration of conditioning pulses in the absence (black circles) and presence (red circles) of 3 mM TCE. Continuous lines represent the best fit of the double exponential function. Each point represents the mean and SEM from seven experiments. **(D)** TCE (3 mM)-induced changes in kinetic parameters [**a**: fast time constant (τ_fast_), **b**: slow time constant (τ_slow_), **c**: amplitude fraction of fast time constant (A_fast_), **d**: amplitude fraction of slow time constant (A_slow_). Each column represents the mean and SEM from seven experiments. **; *p* < 0.01, n.s; not significant. **(E)** TCE (1 mM, 3 mM, and 10 mM)-induced changes in weighted time constant (τ_weighted_) of the P_2_/P_1_ ratio of TTX-R I_Na_. The τ_weighted_ was normalized to the respective control (dotted line). Each column represents the mean and SEM from seven experiments for 1, 3, and 10 mM TCE. *; *p* < 0.05, **; *p* < 0.01, n.s; not significant

We examined the effect of TCE on the onset of inactivation of TTX-R Na^+^ channels using a two-pulse protocol (first conditioning pulse (P_1_): 2–5000-ms duration (up to − 10 mV); second test pulse (P_2_): 100-ms duration (up to − 10 mV) with a 20-ms interpulse interval; Fig. 4A). Figure 4B depicts typical TTX-R I_Na_ elicited by a two-pulse protocol in the absence and presence of 3 mM TCE. We analyzed the amplitude ratio of 2 TTX-R I_Na_ (P_2_/P_1_) to examine the extent of time-dependent inactivation of TTX-R Na^+^ channels. We then calculated the P_2_/P_1_ ratio in the absence and presence of TCE and fitted it to a double exponential function (Fig. 4C). We regarded the two resultant time constants, that is, fast (τ_fast_) and slow (τ_slow_), and their amplitude fractions, that is, fast (A_fast_) and slow (A_slow_), as the kinetic parameters of the onset of inactivation. As shown in Fig. 4D, TCE at a 3 mM concentration decreased the τ_fast_ (69.3 ± 6.1% of that in the control, 140.3 ± 10.1 ms for the control, and 93.9 ± 4.4 ms for 3 mM TCE treatment; *n* = 7, *p* < 0.05); however, this did not affect the τ_slow_, A_fast_, and A_slow_ values. Furthermore, TCE decreased the τ_weighted_ in a concentration-dependent manner. In contrast, at a 3 mM concentration, it decreased the τ_weighted_ (81.2 ± 3.6% of that in the control, 796.8 ± 75.4 ms for the control, and 647.6 ± 70.0 ms for 3 mM TCE treatment; *n* = 7, *p* < 0.01; Fig. 4E).

**Fig. 5:**
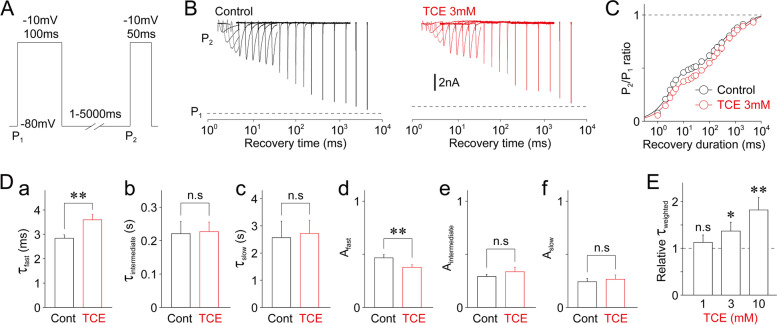
Effect of TCE on the recovery from inactivation of TTX-R Na^+^ channels. **(A)** A schematic illustration of the two-pulse protocol for determining recovery kinetics. The TTX-R I_Na_ was induced by initial conditioning pulses (P_1_: − 10 mV depolarization, 100 ms duration), followed by test pulses (P_2_: − 10 mV depolarization, 50 ms duration). The second TTX-R I_Na_ was recovered with various interpulse intervals of 1–5000 ms at − 80 mV. **(B)** Typical traces of TTX-R I_Na_ elicited by test pulses (P_2_) in the two-pulse protocol in the absence (left) and presence (right) of 3 mM TCE. The dotted lines represent the amplitude elicited by conditioning pulses (P_1_). **(C)** The P_2_/P_1_ ratio of TTX-R I_Na_ against the duration of conditioning pulses in the absence (black circles) and presence (red circles) of 3 mM TCE. Continuous lines represent the best fit of the double exponential function. Each point represents the mean and SEM from seven experiments. **(D)** TCE (3 mM)-induced changes in kinetic parameters [**a**: fast time constant (τ_fast_), **b**: intermediate time constant (τ_intermediate_), **c**: slow time constant (τ_slow_), **d**: amplitude fraction of fast time constant (A_fast_), amplitude fraction of intermediate time constant (A_intermediate_), **f**: amplitude fraction of slow time constant (A_slow_). Each column represents the mean and SEM from seven experiments. **; *p* < 0.01, n.s; not significant. **(E)** TCE (1, 3, and 10 mM)-induced changes in weighted time constant (τ_weighted_) of the P_2_/P_1_ ratio of TTX-R I_Na_. The τ_weighted_ was normalized to the respective control (dotted line). Each column represents the mean and SEM from seven experiments for 1, 3, and 10 mM TCE. *; *p* < 0.05, **; *p* < 0.01, n.s; not significant

We further examined the effect of TCE on the recovery from the inactivation of TTX-R Na^+^ channels using a two-pulse protocol (first conditioning pulse (P_1_): 500-ms duration (up to − 10 mV); second test pulse (P_2_): 100-ms duration (up to − 10 mV) with recovery times at − 80 mV varying from 1 to 5000 ms; Fig. 5A). Figure 5B displays typical TTX-R I_Na_ elicited by a two-pulse protocol in the absence and presence of 3 mM TCE. We analyzed the amplitude ratio of two TTX-R I_Na_ (P_2_/P_1_) to examine the extent of time-dependent recovery of TTX-R Na^+^ channels. We then calculated the P_2_/P_1_ ratio in the absence and presence of TCE and fitted it to a triple exponential function (Fig. 5C). We thus regarded the three resultant time constants, that is, fast (τ_fast_), intermediate (τ_intermediate_), and slow (τ_slow_), and their amplitude fractions, that is, fast (A_fast_), intermediate (A_intermediate_), and slow (A_slow_), as the kinetic parameters of the recovery from inactivation. As shown in Fig. 5D, TCE at a 3 mM concentration increased the τ_fast_ (126.5 ± 2.4% of that in the control, 2.8 ± 0.1 ms for the control, and 3.6 ± 0.2 ms for 3 mM TCE treatment; *n* = 7, *p* < 0.01), whereas decreased A_fast_ (80.9 ± 2.9% of that in the control, 0.47 ± 0.03 for the control and 0.38 ± 0.02 for 3 mM TCE treatment; *n* = 7, *p* < 0.01). However, TCE (3 mM) did not affect the τ_intermediate_, τ_slow_, A_intermediate_, and A_slow_ values (Fig. 5D). Notably, TCE increased the τ_weighted_ in a concentration-dependent manner. In contrast, at a 3 mM concentration, it decreased the τ_weighted_ (137.0 ± 4.8% of that in the control, 671.1 ± 159.2 ms for the control, and 793.2 ± 156.9 ms for 3 mM TCE treatment; *n* = 7, *p* < 0.05; Fig. 5E).

### Effects of TCE on the excitability of small-sized TG neurons

**Fig. 6:**
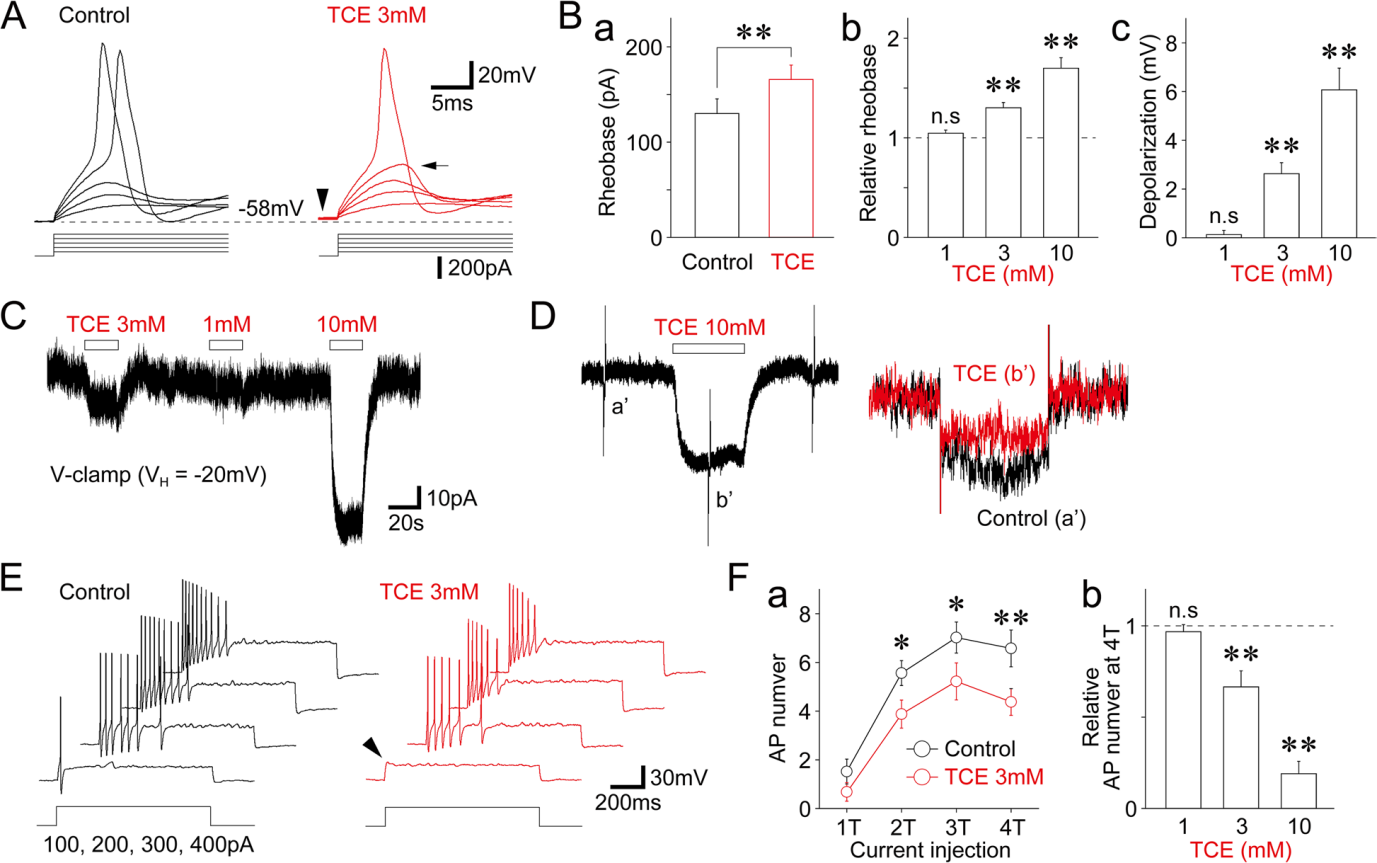
Effect of TCE on the excitability of small-sized TG neurons. **(A)** Typical voltage traces in response to depolarizing current injection in the absence (left) and presence (right) of 3 mM TCE. Five representative raw traces were elicited by successive depolarizing current injection (40–200 pA, 40 pA increment). Note that action potential was not triggered by the fourth stimulation (160 pA injection, arrow), whereas the membrane potential was depolarized (arrowhead) in the presence of 3 mM TCE. **(B) a**, TCE (3 mM)-induced changes in the absolute amplitude of rheobase currents. Each column represents the mean and SEM from eight experiments. **; *p* < 0.01. **b**, TCE (1, 3, and 10 mM)-induced changes in the amplitude of rheobase currents. Each column was normalized to the respective control (dotted line) and represents the mean and SEM from seven, eight, and eight experiments for 1, 3, and 10 mM TCE, respectively. **; *p* < 0.01, n.s; not significant. **c**, TCE (1, 3, and 10 mM)-induced changes in membrane potential. Each column represents the mean and SEM from seven, eight, and eight experiments for 1, 3, and 10 mM TCE, respectively. **; *p* < 0.01, n.s; not significant. **(C)** A typical current trace before, during, and after applying various concentrations of TCE. Small-sized TG neurons were held at a V_H_ of − 20 mM in a voltage-clamp (V-clamp) condition. **(D)** A typical current trace in response to 10-mV hyperpolarizing voltage steps (300-ms duration) before, during, and after applying 10 mM TCE at a V_H_ of − 20 mV. Inset (right) represents typical current traces (a′ and b′) with an expanded time scale. **(E)** Typical voltage traces in response to depolarizing current injection before (left) and during (right) the application of 3 mM TCE. Four representative raw traces were elicited by a one-fold threshold (1T; 100 pA) to the four-fold threshold (4T; 400 pA) depolarizing current injection. Note that the 1T stimulation did not trigger action potentials in the presence of 3 mM TCE (arrowhead). **(F) a**, Changes in the number of action potentials elicited by depolarizing current injection (1T to 4T) in the absence (black circles) and presence (red circles) of 3 mM TCE. Each point represents the mean and SEM from eight experiments. **b**, TCE (1, 3, and 10 mM)-induced changes in the number of action potentials elicited by 4T-depolarizing current injection. Each column was normalized to the respective control (dotted line) and represents the mean and SEM from seven, eight, and eight experiments for 1, 3, and 10 mM TCE, respectively. **; *p* < 0.01, n.s; not significant

We finally examined whether TCE affected the excitability of small-sized TG neurons under the current-clamp conditions. All current-clamp experiments were performed in the presence of 300 nM TTX. In the presence of TTX, the resting membrane potential of small-sized TG neurons was − 55.9 ± 1.8 mV (− 47.8 to − 62.5 mV, *n* = 8). Moreover, under these conditions, TCE increased the rheobase current, which corresponds to the depolarizing threshold current (T) for the generation of the action potential in a concentration-dependent manner. In contrast, at a 3 mM concentration, it increased the rheobase current to 130.2 ± 5.4% of that in the control (130.0 ± 15.3 pA for the control and 165.7 ± 15.1 pA for 3 mM TCE treatment, *n* = 8, *p* < 0.01, Fig. 6A, Ba, Bb). Notably, TCE also depolarized the membrane potential in a concentration-dependent manner (Fig. 6A, Bc). Small-sized TG neurons were held at a V_H_ of − 20 mV in a voltage-clamp condition to further examine the mechanism underlying TCE-induced membrane depolarization. TCE induced inwardly directed currents in a concentration-dependent manner (Fig. 6C), and this TCE-induced change in holding current was accompanied by an increase in input resistance (Fig. 6D). Figure 6E shows typical voltage responses elicited by depolarizing current injection [integers of threshold current (1T to 4T)] in the absence and presence of 3 mM TCE. Notably, TCE significantly decreased the number of action potentials elicited by depolarizing current injection in a concentration-dependent manner. In contrast, at a 3 mM concentration, it decreased the number of action potentials elicited by the 4T current injection to 66.5 ± 8.8% of that in the control (6.6 ± 7.5 for the control and 4.4 ± 5.4 for 3 mM TCE treatment, *n* = 8, *p* < 0.01, Fig. 6E, F).

## Discussion

Reports on the analgesic efficacy of chloral hydrate remain controversial. While chloral hydrate reportedly exhibits substantial analgesic effects [22], a comprehensive review suggests that chloral hydrate has no analgesic properties [7]. Several lines of evidence have suggested that chloral hydrate, through its active metabolite TCE, acts on both the central and peripheral nervous system to elicit analgesic effects. For example, TCE-induced potentiation of central GABA_A_ receptors might contribute to the analgesic effects mediated by chloral hydrate [15, 16], as benzodiazepines, which also potentiate central GABA_A_ receptors, have long been considered primary or adjunctive analgesics [26]. TCE reportedly inhibits P2 × 3 receptors and, to a limited extent, P2Y receptors in nociceptive neurons of the DRG [20], suggesting that TCE-induced inhibition of P2 receptors might contribute to its peripheral analgesic effects. Furthermore, TCE activates nonclassical two-pore-domain K^+^ (K2P) channels, including recombinant or native TREK-1 (KCNK2) and TRAAK (KCNK4) [27–29] at clinically relevant concentrations. Given that these K2P subtypes are also expressed in nociceptive sensory neurons [30, 31] and that the activation of K2P channels, such as TREK-1, is partly responsible for the analgesic efficacy of volatile anesthetics [32, 33], we speculated that K2P channels might be involved in mediating the chloral hydrate-induced analgesic effects.

Our present study provided additional evidence that TCE acts peripherally to exert analgesic effects by inhibiting or modulating TTX-R Na^+^ channels, as these channels play pivotal roles in action potential electrogenesis in nociceptive sensory neurons [3]. We observed that TCE decreased the peak amplitude of TTX-R I_Na_ in a concentration-dependent manner with an IC_50_ value of 18 mM. Considering that the peak plasma concentration of TCE reaches low millimolar concentrations (2–5 mM) after orally administering high doses (> 2.0 g) of chloral hydrate ([14, 34], but see also [13]), TCE at clinically relevant concentrations could elicit small but significant inhibition of TTX-R Na^+^ channels expressed in nociceptive neurons. We also demonstrated that TCE decreased the decay time constant of single TTX-R I_Na_ in a concentration-dependent manner, suggesting that TCE accelerates the deactivation of TTX-R Na^+^ channels. Collectively, these results suggest that chloral hydrate, through its active metabolite TCE, directly acts on peripheral Na^+^ channels to decrease nociceptive transmission. In this context, examining whether TCE has direct inhibitory effects on Na_V_1.7 and Na_V_1.9, TTX-S and TTX-R Na^+^ channel subtypes, expressed in nociceptive sensory neurons bears significance [1].

Notably, I_NaP_ mediated by voltage-gated Na^+^ channels has been implicated in the firing patterns of neurons, such as regular and repetitive firing, in various regions of the brain [35–38]. Furthermore, I_NaP_ is involved in regulating neuronal excitability, including resting membrane potentials, spike initiation, and burst generation of action potentials [39–42]. Moreover, I_NaP_ mediated by TTX-R Na^+^ channels reportedly contributes to the firing frequency of nociceptive sensory neurons [5, 43]. Furthermore, an abnormal increase in I_NaP_ has been implicated in neurological disorders, such as epilepsy and pain, with excessive neuronal excitability [44–46]. When transfected in Na_V_1.8-knockout mouse DRG neurons, human Na_V_1.8 exhibits larger TTX-R I_NaP_ and I_Ramp_ than rat Na_V_1.8 [43]. Furthermore, Na_V_1.8-mediated I_NaP_ along with the firing frequency in response to depolarizing current stimuli are significantly higher in human DRG neurons than in rat DRG neurons [43]. Since the density of TTX-R I_NaP_ or I_Ramp_ is closely related to the firing frequency of nociceptive neurons [5], the pharmacological manipulation of TTX-R I_NaP_ would be a new strategy for the treatment of various pain conditions. In the present study, TCE potently inhibited the TTX-R I_NaP_ and I_Ramp_ with an IC_50_ values of 3.3 mM and 2.0 mM, respectively, and that TCE, even at submillimolar concentrations, slightly but significantly inhibited the non-inactivating currents mediated by TTX-R Na^+^ channels. Therefore, we inferred that the preferential inhibition of TTX-R I_NaP_ by TCE would be responsible for the analgesic efficacy of chloral hydrate.

In addition to the direct inhibition of transient TTX-R I_Na_ and I_NaP_, TCE had multiple effects on various properties of TTX-R Na^+^ channels. First, TCE shifted the voltage-activation and -inactivation relationships toward hyperpolarizing potentials. As voltage dependence is the fundamental property that determines channel activation and inactivation according to the change in membrane potentials [47], TTX-R Na^+^ channels might be further inactivated at resting membrane potentials in the presence of TCE. Second, TCE accelerated the extent of the onset of TTX-R Na^+^ channel inactivation in a concentration-dependent manner. Specifically, TCE decreased τ_fast_ rather than τ_slow_, consistent with an acceleration of the fast rather than slow components of TTX-R Na^+^ channel inactivation. Third, TCE retarded the recovery from the inactivation of TTX-R Na^+^ channels in a concentration-dependent manner. In contrast, it increased τ_fast_ rather than τ_slow_, consistent with the retardation of the fast component of recovery kinetics of TTX-R Na^+^ channels. Finally, TCE increased the extent of use-dependent inhibition of TTX-R Na^+^ channels. As the use-dependent inhibition, as well as the inactivation and recovery kinetics, are directly related to the repetitive generation of action potentials at higher frequencies, TCE might contribute to the inhibition of repetitive action potential generation during sustained depolarization.

Previous studies using a combination of site-specific mutagenesis and patch-clamp technique have revealed the concrete binding sites of TCE to several ion channels. For example, in the GABA_A_ receptors containing α2 and β1 subunits, a specific mutation within transmembrane domain 3 of the β1 subunit (M286W) abolishes TCE-induced potentiation of Cl^−^ current [16]. In contrast, low concentrations of TCE do not enhance the Cl^−^ current mediated by glycine receptors with two specific mutations α1 subunit (S267I and A288W) [16]. The TCE sensitivity to NMDA receptors is also altered by specific mutations in the GluN2A subunit (F637W and A825W) [48]. Finally, the positive modulation of 5-HT3A receptors by TCE is dramatically reduced by the R246A mutation [49]. At this stage, however, the mechanism by which TCE inhibits and modulates various properties of TTX-R Na^+^ channels remains unknown. As TCE is a lipophilic compound with an octanol/buffer partition coefficient of 1.42, it is expected to incorporate hydrophobic compartments, such as lipid membrane, with the subsequent hydrophobic interactions with channel proteins being potentially responsible for the modulation of TTX-R Na^+^ channels. A simulation study suggested that TCE could bind to protein targets with higher affinities, suggesting that it might bind to ion channels during membrane partition [50]. This molecular behavior is similar to that of volatile anesthetics, such as isoflurane and sevoflurane, which exhibit a distinct preference for the membrane interface [51]. However, the possibility that TCE directly interacts with the outer vestibule of TTX-R Na^+^ channels cannot be excluded. Considering that lysine 806 in domain II of Na_V_1.8 plays a role in the high sensitivity of Na_V_1.8 to open-channel blockage by *n*-alcohol [52], a combination of site-specific mutagenesis and patch-clamp technique may reveal the detailed binding sites and mechanisms underlying TCE-mediated modulation of TTX-R Na^+^ channels.

In the present study, TCE increased the amplitude of rheobase current to generate action potentials but decreased the number of action potentials elicited by depolarizing current stimuli. Considering that I_NaP_ plays crucial roles in the repetitive generation of action potentials in central neurons within various brain regions [38–42, 53] and that TTX-R I_NaP_ contributes to the excitability of nociceptive sensory neurons [5], we speculated that the preferential inhibition of TTX-R I_NaP_ mediates TCE-induced changes in the number of action potentials. The modulation of inactivation and recovery kinetics and use-dependent inhibition of TTX-R Na^+^ channels might be additionally responsible for the TCE-induced changes in neuronal excitability. We previously reported that TTX-R I_NaP_ is closely related to the inactivation and recovery kinetics and use-dependent inhibition of TTX-R Na^+^ channels in nociceptive sensory neurons [5]. Furthermore, we also observed that TCE slightly depolarized the membrane potential. This TCE-induced membrane depolarization might be mediated by the inhibition of leak K^+^ channels, such as K2P channels because the TCE-induced decrease in holding current was accompanied by an increase in input resistance. However, as TCE at a higher concentration (10 mM) exhibited significant changes in input resistance, the effect of TCE at a clinically relevant concentration on input resistance would be negligible.

## Conclusion

In conclusion, we demonstrated that TCE, an active metabolite of chloral hydrate, inhibited the TTX-R I_Na_ and modulated various properties of these channels at clinically relevant concentrations, resulting in decreased excitability of nociceptive neurons. These pharmacological characteristics provide novel evidence for the analgesic efficacy mediated by chloral hydrate in pediatric dentistry.

## Data Availability

The datasets used and/or analysed during the current study are available from the corresponding author on reasonable request.

## Abbreviations

DRG: dorsal root ganglia
I_Na_: Na^+^ current
I_NaP_: persistent I_Na_
I_Na__T_: transient I_Na_
I_Ramp_: slow voltage-ramp-induced current
TCE: 2′,2′,2′-trichloroethanol
TG: trigeminal ganglia
TTX-R: tetrodotoxin-resistant
TTX-S: tetrodotoxin-sensitive
